# Regulation of MicroRNA-497-Targeting AKT2 Influences Tumor Growth and Chemoresistance to Cisplatin in Lung Cancer

**DOI:** 10.3389/fcell.2020.00840

**Published:** 2020-09-08

**Authors:** Lin Wang, Xiang-Bo Ji, Li-Hong Wang, Jian-Ge Qiu, Feng-Mei Zhou, Wen-Jing Liu, Di-di Wan, Marie Chai-mi Lin, Ling-Zhi Liu, Jian-Ying Zhang, Bing-Hua Jiang

**Affiliations:** ^1^The First Affiliated Hospital of Zhengzhou University, Zhengzhou University, Zhengzhou, China; ^2^BGI College & Henan Institute of Medical and Pharmaceutical Sciences, Zhengzhou University, Zhengzhou, China; ^3^Department of Oncology, The Affiliated Cancer Hospital of Zhengzhou University (Henan Cancer Hospital), Zhengzhou, China; ^4^Department of Pathology, Anatomy and Cell Biology, Thomas Jefferson University, Philadelphia, PA, United States

**Keywords:** miR-497, AKT2, tumor growth, chemoresistance, NSCLC

## Abstract

**Background:**

MicroRNA-497 (miR-497) has been implicated in several cancers. Increasing studies demonstrate the role of AKT2 in cancers as an oncogene which is closely associated with tumor aggressiveness by enhancing cancer cell survival, migration and invasion However, miR-497/AKT2 axis in non-small cell lung cancer (NSCLC) remains unclear.

**Methods:**

Quantitative real-time PCR (qRT-PCR) was used to quantify the expression of miR-497 and its target gene. The function of miR-497 in lung cancer was investigated through *in vitro* and *in vivo* assays (cell proliferation assay, cell migration assay, colony formation assay, flow cytometry assay, immunoblotting and tumorigenesis assay). Luciferase reporter assay was conducted to confirm the target gene of miR-497.

**Results:**

In this study, we found that miR-497 was significantly downregulated in tumor tissues and blood samples of lung cancer patients. To understand the potential mechanism of miR-497 in inhibiting tumor growth, we showed that miR-497 blocked the activation of AKT2 and regulated cell proliferation, cell migration, colony formation and increases chemosensitivity of H1299 cells to cisplatin by inhibiting AKT2. MiR-497 also inhibited tumor growth and suppressed expression of AKT2 at the protein and mRNA levels in mouse xenograft tumors.

**Conclusion:**

Taken together, our findings indicated that miR-497 suppresses the tumor growth by targeting AKT2, and the miR-497/AKT2 axis is a potential therapeutic target for NSCLC intervention.

## Introduction

Lung cancer in the world is the leading cause of cancer-related mortality, much progress has been made in the treatment of lung cancer in the past several years. Non-small cell lung cancer (NSCLC) accounts for approximately 85% of lung cancer cases (Molina et al., 2008). Cisplatin (CDDP)-based chemotherapy is widely used as the first-line chemotherapeutic agent for advanced NSCLC (Scagliotti et al., 2008; Tsai et al., 2011). However, patients can acquire resistance to CDDP treatment in 6 months to 1 years, the mechanisms underlying NSCLC and chemoresistance remain unknown. Better investigation of the underlying mechanisms in tumor development and chemoresistance in NSCLC would be indispensable in promoting prevention, clinical diagnosis and treatment.

MicroRNAs are a group of small 19–25 nucleotides (∼22 nt) non-coding regulatory RNA molecules (Calin and Croce, 2006; Liu et al., 2011; Xu et al., 2012). Recently, microRNAs have been implied in major cellular pathways to regulate cell differentiation, proliferation, survival and tumorigenesis (Pasquinelli, 2012; Xu et al., 2013; Jiang et al., 2018; Lin et al., 2019). MiR-497 downregulation has been known in various cancer, which is associated with tumor growth. Current known miR-497 targets are CCNE1, VEGFA, Bcl2, KSR1, HDGF and HIF1α, which are involved in tumorigenesis of various cancers (Han et al., 2015; Wei et al., 2015; Qiu et al., 2016; Wang et al., 2016; Wu D. et al., 2016; Wu Z. et al., 2016). However, role of miR-497 in lung cancer is still unclear.

AKT2, an isoform of AKT family, is reported to be a significant member of PI3K/AKT pathway (Cheng et al., 1992; Nicholson and Anderson, 2002). Recent studies demonstrated that AKT2 is an oncogene associated with tumor aggressiveness through promoting cancer cell survival and invasion (Arboleda et al., 2003; Cheng et al., 2007). In this study, AKT2 oncogene has been validated as a novel target of miR-497. Here we also showed that miR-497 inhibited cell proliferation, cell migration, colony formation and chemoresistance via its target AKT2 expression.

In this study, we would address following questions: (a) whether miR-497 expression associated with lung cancer incidence; (b) what a new target of miR-497 is involved in lung cancer; (c) what is the underlying role of miR-497 in tumor growth; and (d) whether miR-497 induce chemosensitivity to CDDP treatment in lung cancer. This study will questions to classify the roles and mechanisms of miR-497 in lung cancer tumorigenesis, be useful for developing new miR-497/AKT2 -based prognostic marker or/and treatment option.

## Materials and Methods

### Human Clinical Specimens

Cancer tissues and adjacent normal tissues were collected from lung cancer patients undergoing surgical procedure. Parts of the samples were fixed in formalin for histological examination, and parts were immediately snap-frozen in liquid nitrogen. All clinical samples were histologically classified and de-identified based on diagnosis using the CoPath Anatomic Pathology system. No patient information regulated by HIPPA was available for this study. Experiment protocols have been approved by the Ethics Committees of Zhengzhou University.

Human blood samples (No patient information regulated by HIPPA were available for this study) were collected from Henan Cancer Hospital and The First Affiliated Hospital of Zhengzhou University with consent of patients and healthy subjects. Experiment protocols were approved by Ethics Committees of Zhengzhou University. Plasma samples were de-identified based on diagnosis using the CoPath Anatomic Pathology system (no patient information is available), and isolated from bloods by centrifuging at 3,500 rpm for 10 min, then 300 μL of plasma and 900 μL of TRIZol reagent were thoroughly mixed, *C. elegans miR-39* (*cel-miR-39*) was added to final concentration of 10^–5^ nM as spiked-in control. According to the manufactory’s instruction, RNA were extracted and analyzed by qRT-PCR.

### Cell Culture and Stable Cell Line Establishment

Human lung cancer cells H1299, A549, and H1975 were cultured in RPMI 1640 medium, and HEK293T were maintained in DMEM in a 37°C incubator containing 5% CO_2_. To stably overexpress miR-497 in lung cancer cells, following the manufacturer’s manual, lentivirus carrying miR-497 or negative control (miR-NC) were packaged with HEK293T cells (Thermo Fisher Scientific, Huntsville, AL, United States).

### Cell Viability Assay

Indicated amount of cells were seeded in 96-well culture plates. According to the manufacturer’s instruction, CCK8 kit was used to test the cell viability (Dojindo Laboratories, Japan). Results were obtained with five replicates per experiment from three separate experiments.

### *In vitro* Migration Assay

Migration assay were conducted with BD migration chambers according to manufacturer’s instruction (BD Biosciences, United Kingdom). Cells (without serum) were seeded in the upper chamber. Then lower chamber were filled with RPMI 1640 medium (with serum). Experiments were independently repeated three times.

### Colony Formation Assay

Cells were placed in 12-well plates, and medium was replaced every week. Almost 14 days later, cells were fixed and stained with crystal violet (Sigma-Aldrich). Visible colonies were counted and measured. Experiments were independently repeated three times.

### Dual-Luciferase Reporter Assay

AKT2 (3’-UTR fragment) was amplified by PCR using Pfu DNA polymerase from cDNA library of H1299 cells. The PCR products were inserted into pMIR-REPORTER vector. Wild type (WT) and mutant (MT) constructs were confirmed by DNA sequencing. Cells were cultured and co-transfected with luciferase reporter plasmids, renilla luciferase reporter (internal control) and equal amounts of miR-NC or miR-497. 24–48 h later, dual-luciferase (Firefly and Renilla luciferase) activities were measured (Promega, WI, United States). Experiments were independently repeated three times.

### RNA Isolation and qRT-PCR Analysis

In accordance with manufacturer’s instruction, total RNAs from human specimens or cultured cells were extracted with TRIzol reagent (Invitrogen, CA, United States). To quantify AKT2 mRNA levels, oligo dT primer were used for transcription with RT Kit (Vazyme, China). GAPDH levels were used as an internal control. To measure expression levels of miR-497, stem-loop RT primers were used for transcription with RT Kit (Vazyme, China). SYBR Green Mix (Vazyme, China) was used for qRT-PCR on a 7900HT system. U6 levels were used as an internal control.

### Immunoblotting

Human tissues or cells lysates were harvested with RIPA buffer, then centrifugated at 12,000 rpm for 20 min, and supernatants were collected. SDS-polyacrylamide gel electrophoresis (SDS-PAGE) were used for Immunoblotting. The gels were transferred onto nitrocellulose membranes with transfer buffer. 5% non-fat dry milk were used to block membranes, and then incubated with primary antibodies against AKT2 (Proteintech, United States), GAPDH (Bioworlde, Atlanta, United States). ECL Detection System was used for signal detection (Thermo Scientific, Rockford, IL, United States).

### *In vitro* Chemosensitivity Array

5,000 cells were seeded per well in a 96-well plate for 24 h. Then, freshly prepared CDDP (Sigma-Aldrich, United States) was added into medium, finally to obtain indicated concentrations (2.5, 5, 10, 20, 40, 80 to 160 μM). 48 h later, CCK8 kit was used to value the cell viability. Experiments were independently repeated three times.

### Apoptosis Assay

Apoptosis assay was measured by Annexin V staining. Flow cytometry (FACS Canto II, BD Biosciences) was used for analysis of cell apoptosis rate. The data were analyzed using FlowJo software. Experiments were independently repeated three times.

### Caspase-3 Activity Assay

Caspase-3 activity was determined with caspase-3 activity kit (Beyotime, China). According to the manufacturer’s protocol, cell lysates after treatment were harvested and incubated with reaction buffer which containing Ac-DEVD-pNA (caspase-3 substrate). Absorbance at 405 nm was used to measure the reaction.

### Xenograft Studies

Female nude mice (BALB/cA-nu) were purchased from Laboratory Animal Center (Shanghai, China), and maintained in SPF (special pathogen-free) conditions. For tumor growth assay, protocols were approved by Animal Welfare Committee of Zhengzhou University. 5 × 10^6^ cells were injected into posterior flank of nude mice. Tumor when they were visible was measured using vernier caliper every two days, and tumor volume was calculated according to the formula (volume = 0.5 × Length × Width^2^).

### Statistical Analysis

Data were performed as means ± SD of 3 independent experiments. Student’s unpaired *t* test in this study was used for comparison of 2 independent groups. Correlations between miR-497 and AKT2 levels in human NSCLC tissues were analyzed by Pearson’s rank test. Values were considered significantly different at *P* < 0.05.

## Results

### Down-Regulation of miR-497 Expression in Human Blood Samples and Lung Cancer Tissues

**FIGURE 1:**
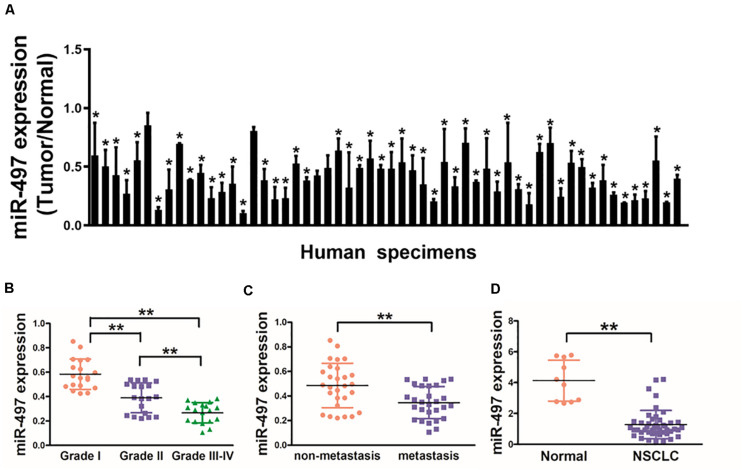
Down-regulation of miR-497 expression in tumor tissues of human lung cancer patients. **(A)** Expression levels of miR-497 in 56 pairs of lung cancer tumor and adjacent normal specimens were analyzed by stem-loop qRT-PCR, and normalized to the levels of U6. The fold changes were obtained by the ratio of miR-497 abundance in cancer tissues to that in the adjacent normal tissues. **(B)** Relative expression levels of miR-497 in different stages of cancer tissues. **(C)** qRT-PCR analysis of miR-497 expression in 56 pairs of primary NSCLC tissues and their corresponding lymph node metastases. **(D)** miR-497 expression was decreased in plasma of NSCLC patients. The relative expression levels of miR-497 were detected and normalized to the spiked-in control cel-miR-39 (spiked-in control). Data were presented by mean ± SD. of 3 replicates. *indicated *P* < 0.05, ** indicated *P* < 0.01.

In this study, we tested the expression levels of miR-497 in 56 pairs of NSCLC and normal tissues, which as showed in Figure 1A that expression levels of miR-497 in NSCLC tissues were significantly lower than normal tissues. Moreover, miR-497 expression levels were significantly lower in Grade III-IV tissues compared with those in Grade I/II, which indicating that miR-497 was significantly down-regulated in late stages of lung cancer (Figure 1B). Interestingly, when 56 NSCLC samples were classified on the basis of the status of lymph node, we observed miR-497 were significantly lower in NSCLC with lymph node spread than those without tissues (Figure 1C). Human plasma samples showed that miR-497 were markedly decreased in 46 NSCLC patients compared with 10 healthy subjects, which suggested that miR-497 expression levels can be detected in the circulation blood samples as a potential biomarker (Figure 1D). Taken together, the low expression levels of miR-497 in blood samples and tumor tissues of NSCLC patients could be used as a potential new biomarker for the diagnosis and advancement of NSCLC.

### AKT2 Is a Direct Target of miR-497

**FIGURE 2:**
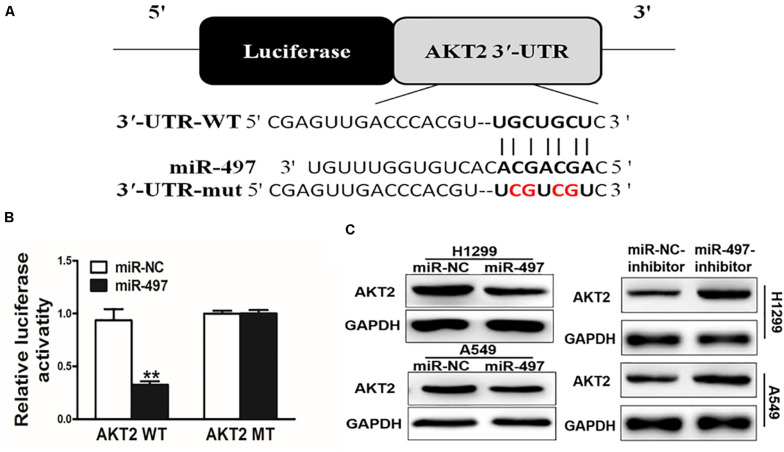
AKT2 is a direct target of miR-497. **(A)** Putative seed-matching sites or mutant sites (red) between miR-497 and 3’-UTR of AKT2. **(B)** Reporter assay of miR-497 targeting AKT2. Luciferase activities of reporter constructs containing wild-type (WT) or mutant (MT) 3’-UTR of AKT2 were assayed and normalized to those of renilla activities (internal control). **(C)** Suppression of AKT2 protein levels by miR-497. Total proteins were subjected to western blotting and detected for AKT2 expression levels.

We searched for the probable targets of miR-497 using the combination of PicTar, TargetScan, and KeyTar algorithms, and found that there was a putative miR-497 binding site in 3′-UTR region of AKT2. To test whether miR-497 targets AKT2, as indicated in Figure 2A, we respectively cloned 3′-UTR region of AKT2 and the mutated sequences into pMIR-REPORTER vector. Overexpression of miR-497 significantly decreased reporter activities by almost 60%, while it did not affect the mutant reporter luciferase activities, suggesting that the mutation site was the binding regions of miR-497 and AKT2 3′-UTR (Figure 2B). Immunoblotting showed that miR-497 overexpression was sufficient to inhibit AKT2 protein expression (Figure 2C and Supplementary Figure S1). Moreover, inhibition of miR-497 induced AKT2 protein expression (Figure 2C). These results demonstrated that miR-497 directly inhibited AKT2 expression by directly targeting its 3′-UTRs.

### Lung Cancer Tissues Exhibits Higher Levels of AKT2 Associated With miR-497 Downregulation

**FIGURE 3:**
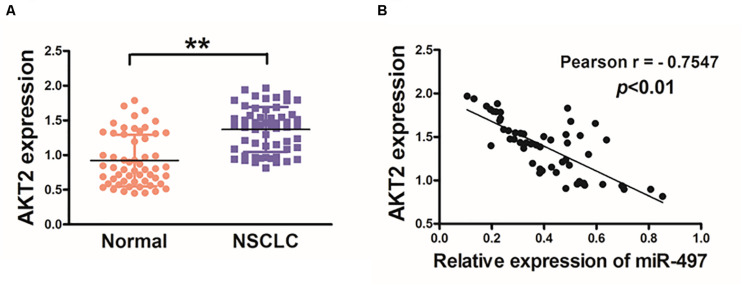
Lung cancer tissues exhibits higher levels of AKT2 which are inversely correlated with miR-497 expression. **(A)** The expression levels of AKT2 in normal tissues and human NSCLC specimens were determined by qRT-PCR analysis and fold changes were obtained by the ratios of AKT2 to GAPDH levels. **(B)** Pearson’s correlation analysis was used to determine the correlation between the expression levels of AKT2 and miR-497 in human NSCLC specimens. Data were presented by mean ± SD. of 3 replicates. **indicated *P* < 0.01.

Then, we investigated AKT2 levels in human cancer tissues, and higher mRNA levels of AKT2 were found in NSCLC tissues compared with adjacent non-tumor tissues (Figure 3A). We further investigated the correlation between AKT2 and miR-497 expression levels in NSCLC tissues. As shown in Figure 3B, Pearson’s correlation analysis showed that AKT2 levels in NSCLC samples were negatively correlated with miR-497 expression levels (Pearson’s correlation r = −0.7547, *p* < 0.01).

### MiR-497 Overexpression Inhibits Cell Proliferation, Cell Migration and Colony Formation, Overexpression of AKT2 Reverses the Inhibitory Effects of miR-497

**FIGURE 4:**
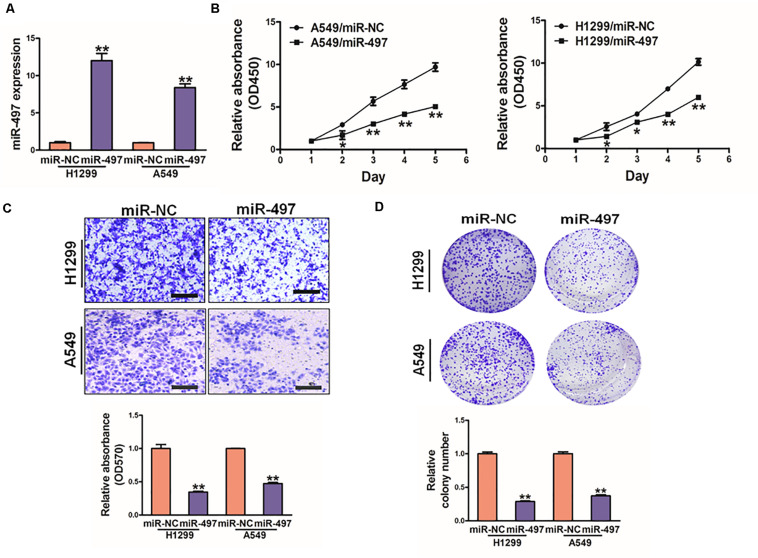
MiR-497 overexpression suppresses cell proliferation, migration and colony formation of lung cancer cells. **(A)** Expression levels of miR-497 was conducted in A549 and H1299 cells. **(B)** Overexpression of miR-497 decreased cell growth activity. **(C)** Migration assay of A549 and H1299 cells. The results showed that overexpression of miR-497 inhibited cell migration. **(D)** Colony formation was conducted in indicated cells, while the activity of colony formation were reduced in miR-497 group compared with miR-NC. Data were presented by mean ± SD. of 3 replicates. *indicated *P* < 0.05, **indicated *P* < 0.01.

To further study direct role of miR-497 in lung cancer, we established stable cell lines (A549 and H1299 cells) with lentivirus carrying miR-497 or negative control (miR-NC). The stable cell lines were confirmed by high expression levels of miR-497 (Figure 4A). CCK8 kit indicated that miR-497 overexpression significantly reduced cell proliferation rate 48h after the seeding (Figure 4B and Supplementary Figure S1). Overexpression of miR-497 also significantly decreased the activity of cell migration (Figure 4C). Then colony formation assay was conducted in indicated cells, which showed that miR-497 overexpression reduced the activity of colony formation (Figure 4D, Supplementary Figure S1). Moreover, inhibition of miR-497 in H1299 induced cell proliferation and colony formation activity (Supplementary Figure S2). These results indicated that the miR-497 overexpression is sufficient to suppress cell proliferation, cell migration and colony formation.

**FIGURE 5:**
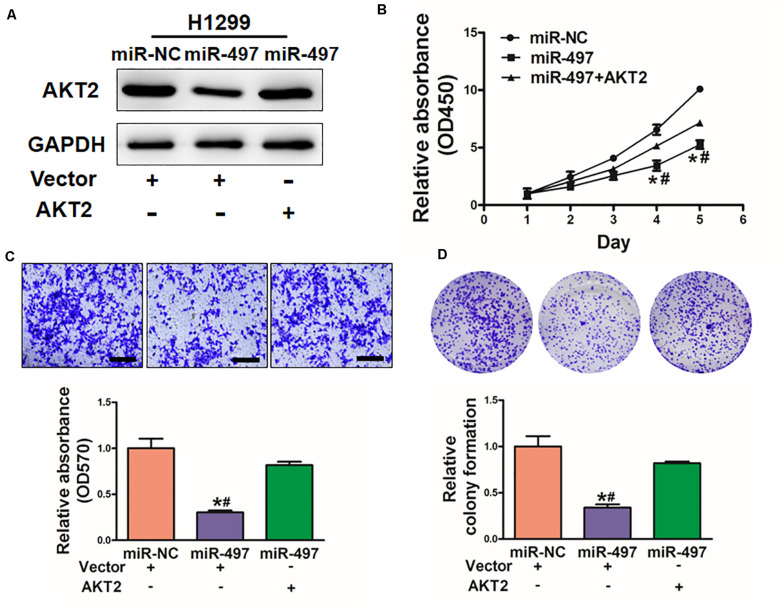
Overexpression of AKT2 reverses the inhibitory effects of miR-497. **(A)** The expression of AKT2 was rescued by transfection of pCMV-AKT2 for 48 h. **(B)** Overexpression of miR-497 arrested cell proliferation, but this was rescued upon coexpression of exogenous AKT2 in H1299 cells. **(C)** MiR-497 overexpression decreased cell migration by its target AKT2 in H1299 cells. **(D)** Overexpression of AKT2 restored miR-497-inhibited colony formation. Data were presented by mean ± SD of 3 replicates. *or ^#^ indicated *P* < 0.05. *indicates significant difference compared to control; ^#^indicates significant difference compared to miR-497 treatment plus AKT2.

To explore whether miR-497 exerts its function through AKT2, H1299 cells were co-transfected with miR-497 or miR-NC together with pCMV-AKT2 for 48 h (Figure 5A). Overexpression of AKT2 lacking the miR-497- targeting 3′-UTR rescued the inhibition effect of miR-497 in cell proliferation and cell migration (Figures 5B,C). Similarly, AKT2 overexpression rescued miR-497-inhibited colony formation (Figure 5D). These findings suggested that miR-497 inhibits activity of cell proliferation, cell migration and colony formation through targeting AKT2.

### MiR-497 Sensitizes Lung Cancer Cells to CDDP Treatment by Suppressing AKT2

**FIGURE 6:**
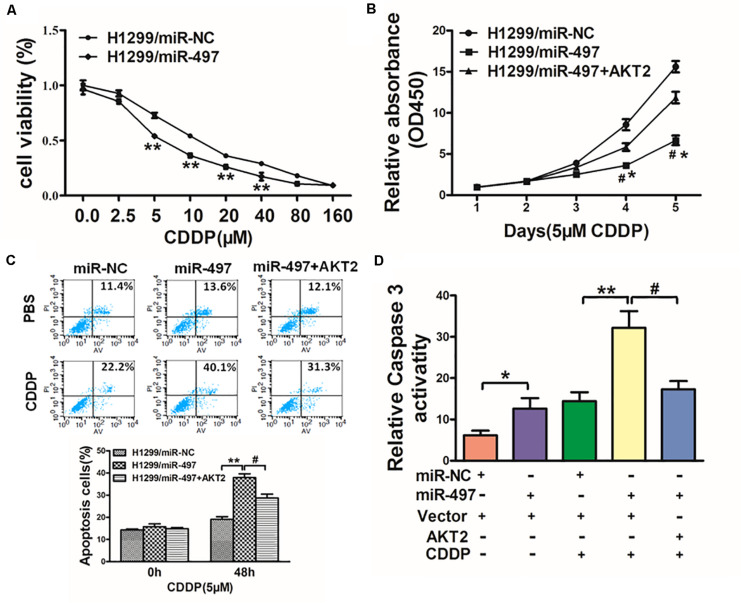
MiR-497 sensitizes lung cancer cells to CDDP treatment by suppressing AKT2. **(A)** H1299 cells stably expressing miR-NC or miR-497 were treated with different concentrations of CDDP for 48 h, and cell viability was analyzed using CCK-8 assay. **(B)** H1299 cells stably expressing miR-NC, miR-497, or miR-497 in combination with AKT2 overexpression were treated with 5 μM of CDDP for indicated time points. Cell viability was analyzed by CCK-8 assay. **(C,D)** Cell apoptosis was analyzed by flow cytometry and by caspase-3 assay. Data represent mean ± SD from three replicates. *, ^#^indicates *P* < 0.05; **indicates *P* < 0.01, *indicates *P* < 0.05 compared to miR-NC control. ^#^indicates *P* < 0.05 compared to miR-497 and AKT2 overexpression group.

Chemoresistance to CDDP is still one of the main causes of drug resistance in human lung cancer. Thus, it is essential to discover new strategies to increase CDDP effectiveness. We showed with H1299 cells as indicated in Figure 6A that forced expression of miR-497 significantly increased sensitivity to CDDP. Cell viability rate of 5 μM CDDP treatment were measured by CCK-8 assay at different time points, which showed that overexpression of AKT2 rendered cancer cells more chemoresistance in miR-497-overexpressing lung cancer cells (Figure 6B). To deeply study whether miR-497/AKT2 functioned in cell apoptosis with CDDP, we conducted apoptosis analysis and caspase-3 assay. Resultly, miR-497 plus CDDP significantly induced cell apoptosis, overexpression of AKT2 partially abolished the miR-497-inducing apoptotic effect (Figure 6C). We then showed that the activity of caspase-3, act as main executor of cell apoptosis, was significantly increased in miR-497 plus CDDP compared with miR-497 or CDDP treatment alone, overexpression of AKT2 attenuated caspase-3 induction (Figure 6D). These results demonstrated that miR-497 renders cancer cells more sensitive to CDDP, while AKT2 overexpression reversed effect of miR-497 and CDDP in H1299 cells.

### MiR-497 Inhibits Tumor Growth *in vivo*

**FIGURE 7:**
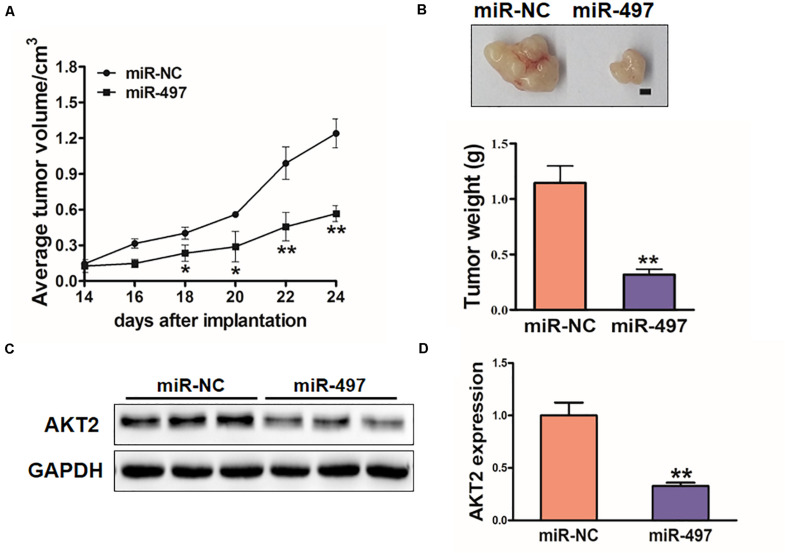
MiR-497 inhibits tumorigenesis *in vivo*. **(A,B)** Tumor growth assay in nude mice. Tumor growth curve, representative pictures and average weight of xenograft tumors between the groups of miR-NC and miR-497. Bar: 2 mm. **(C)** Protein levels of AKT2 in xenograft tumors. **(D)** The expression levels of AKT2 were analyzed by qRT-PCR. Data were presented by mean ± SD. of 3 replicates. *indicated *P* < 0.05; **indicates *P* < 0.01.

To investigate the *in vivo* effect of miR-497, H1299 stable cell lines were subcutaneously injected into female nude mice (*n* = 6). 18 days after post-implantation, tumor volumes of miR-497 overexpressed group were showed significantly smaller (Figure 7A). Nude mice were sacrificed on Day 26 after the injection, and xenografts were collected. Figure 7B showed the representative xenograft tumors, which indicated that average tumor weights of miR-497 group was decreased by 70%. Total RNAs and proteins in tumor samples were analyzed by qRT-PCR and Immunoblotting, which showed that miR-497 repressed expression of AKT2 in tumor tissues (Figures 7C,D).

## Discussion

Recently, miRNAs are reported to secret into blood system by normal cells and/or tumor cells, that may be used as biomarkers (Detassis et al., 2017). Levels of several different miRNAs in blood are found to be altered in some cancers (Moretti et al., 2017; Zhou Q. et al., 2018; Zhou X. et al., 2018). MicroRNA-497 has been reported to inhibit thyroid cancer tumor growth and invasion by suppressing BDNF (Wang et al., 2017), which is involved in cell proliferation and invasion of gastric cancer by repressing eIF4E (Li et al., 2014), and to inhibit colorectal cancer growth by targeting insulin-like growth factor 1 receptor (Guo et al., 2013). In this study, we initially showed that miR-497 inhibits cell growth, migration, colony formation; and reverses chemoresistance in lung cancer cells.

AKT2 was known as an oncogene to promote cancer development (Jiang et al., 2016). In this study, AKT2 has been validated as a new target of miR-497 *in vitro* and *in vivo*. Higher mRNA levels of AKT2 were found in NSCLC tissues than non-tumor tissues with an inverse correlation between AKT2 and miR-497 expression levels in human tumor samples. This study firstly provides the evidence that miR-497 is important in abating lung cancer tumorigenesis through inhibiting AKT2 translation.

Recent studies showed that acquired drug resistance in cancer cells might be regulated by alterations in miRNA levels (Hummel et al., 2010; Ma et al., 2010; Zheng et al., 2010; Ge et al., 2018). We have demonstrated that miR-143 inhibits tumor growth of colorectal cancer and angiogenesis (Qian et al., 2013), which makes cells more chemosensitivity to oxaliplatin treatment, and miR-218 repression contributes to progression of EMT (epithelial-mesenchymal transition), chemoresistance and tumor metastasis by targeting Slug/ZEB2 signaling in lung cancer (Shi et al., 2017). Other study suggested that cell stemness and chemoresistance via targeting DLK1 (Ma et al., 2017). In this study, we showed that miR-497 promotes H1299 cells more sensitive to CDDP, these results indicate that microRNAs play an important role in chemoresistance.

Alterations of miRNAs functioned in tumorigenesis and cancer progression including lung cancer (Khoshgoo et al., 2017; Krentz Gober et al., 2017). Present investigation suggested that miR-497 acts as a tumor suppressor, negatively regulates AKT2 oncogene via its 3′-UTR. In addition, we demonstrate that miR-497 sensitizes lung cancer cells to CDDP treatment in an AKT2-dependent manner. Taken together, we showed that miR-497/AKT2 may be potential new lung cancer biomarkers, which would be interesting for further investigation.

## Conclusion

In summary, this study suggested that miR-497/AKT2 regulatory axis in lung cancer, which miR-497 negatively regulates AKT2 oncogene expression and functions as a tumor suppressor. MiR-497 highlighted its role and sensitized lung cancer cells to CDDP treatment in an AKT2-dependent manner. These findings may provide potential new biomarkers for lung cancer diagnosis, prevention and treatment.

## Data Availability Statement

The original contributions presented in the study are included in the article/Supplementary Material, further inquiries can be directed to the corresponding author/s.

## Ethics Statement

The studies involving human participants were reviewed and approved by Ethics Committees of Zhengzhou University. The patients/participants provided their written informed consent to participate in this study. The animal study was reviewed and approved by Animal Welfare Committee of Zhengzhou University.

## Author Contributions

LW, X-BJ, and L-HW performed experiments, analyzed data, and wrote the manuscript. J-GQ, W-JL, DW, F-MZ and ML performed qRT-PCR assay, reporter assay and immunoblotting analysis, analyzed chemosensitivity array and animal analysis. LW, L-ZL, J-YZ and B-HJ designed the project andrevised the manuscript. All authors approved the submitted version.

## Conflict of Interest

The authors declare that the research was conducted in the absence of any commercial or financial relationships that could be construed as a potential conflict of interest.

## Abbreviations

AKT2: v-AKT murine thym‘oma viral oncogene homolog 2
3,-UTR: 3: untranslated region
PI3K/AKT: phosphoinositide 3-kinase/serine/threonine kinase 1
HIF-1 α: hypoxia-inducible factor-1 α
MiR-497: microRNA-497
NSCLC: non-small cell lung cancer
SPF: special pathogen-free.
