# Corrigendum: Regulation of microrna-497-targeting AKT2 influences tumor growth and chemoresistance to cisplatin in lung cancer

**DOI:** 10.3389/fcell.2022.1008088

**Published:** 2022-09-23

**Authors:** Lin Wang, Xiang-Bo Ji, Li-Hong Wang, Jian-Ge Qiu, Feng-Mei Zhou, Wen-Jing Liu, Di-di Wan, Marie Chai-mi Lin, Ling-Zhi Liu, Jian-Ying Zhang, Bing-Hua Jiang

**Affiliations:** ^1^ The First Affiliated Hospital of Zhengzhou University, Zhengzhou University, Zhengzhou, China; ^2^ BGI College & Henan Institute of Medical and Pharmaceutical Sciences, Zhengzhou University, Zhengzhou, China; ^3^ Department of Oncology, The Affiliated Cancer Hospital of Zhengzhou University (Henan Cancer Hospital), Zhengzhou, China; ^4^ Department of Pathology, Anatomy and Cell Biology, Thomas Jefferson University, Philadelphia, PA, United States

**Keywords:** MiR-497, Akt2, tumor growth, chemoresistance, NSCLC

In the published article, there was a mistake in the **Author Contributions**. The author L-ZL was accidentally included in the contribution, “performed qRT-PCR assay, reporter assay and immunoblotting analysis, analyzed chemosensitivity array and animal analysis”, and for authors LW, L-ZL, J-YZ, and B-HJ the contribution “designed the project and revised the manuscript” was accidentally omitted.

The correct Author Contributions appear below:

“LW, X-BJ, and L-HW performed experiments, analyzed data, and wrote the manuscript. J-GQ, W-JL, DW, F-MZ, and ML performed qRT-PCR assay, reporter assay and immunoblotting analysis, analyzed chemosensitivity array and animal analysis. LW, L-ZL, J-YZ, and B-HJ designed the project and revised the manuscript. All authors approved the submitted version.”

The authors apologize for this error and state that this does not change the scientific conclusions of the article in any way. The original article has been updated.

