# Supplementary material for: Regulation of MicroRNA-497-Targeting AKT2 Influences Tumor Growth and Chemoresistance to Cisplatin in Lung Cancer
Source: Front Cell Dev Biol. 2020 Sep 8;8:840. doi: 10.3389/fcell.2020.00840 (PMC7505950; doi:10.3389/fcell.2020.00840)

Supplementary data

Sup Figure 1. MiR-497 overexpression suppresses cell proliferation, colony formation and AKT2 expression of H1975 lung cancer cell. (A) Overexpression of miR-497 decreased cell growth activity. **(B)** Colony formation was conducted in indicated cells, while the activity of colony formation were reduced in miR-497 group compared with miR-NC. **(C)** AKT2 protein levels was suppressed by miR-497. Data were presented by mean±SD. of 3 replicates. * indicated *P*<0.05. ** indicated *P*<0.01.

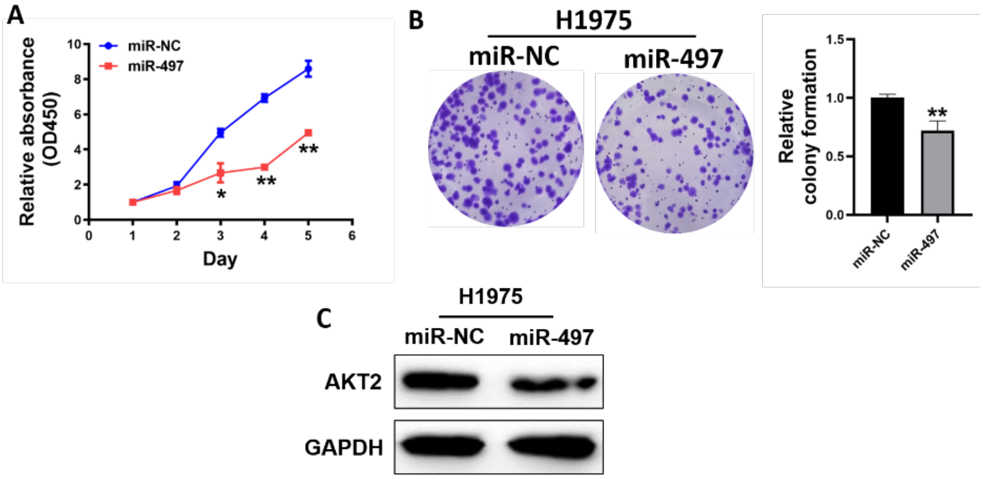

Sup Figure 2. Inhibition of miR-497 in H1299 induced cell proliferation and colony formation activity. (A) Inhibition of miR-497 increased cell growth activity. **(B)** Colony formation was conducted, and the activity of colony formation were induced in miR-497-inhibitor group. Data were presented by mean±SD. of 3 replicates. * indicated *P*<0.05. ** indicated *P*<0.01.

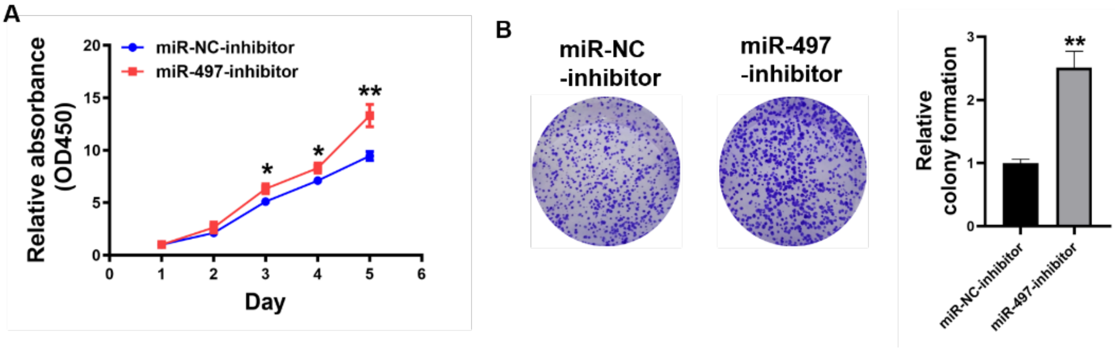

Supplement: Supplementary file 1 [file Image_1.pdf]
